# Three-Dimensional Kidney-on-a-Chip Assessment of Contrast-Induced Kidney Injury: Osmolality and Viscosity

**DOI:** 10.3390/mi13050688

**Published:** 2022-04-28

**Authors:** Kipyo Kim, Beomgyun Jeong, Yun-Mi Lee, Hyung-Eun Son, Ji-Young Ryu, Seokwoo Park, Jong Cheol Jeong, Ho Jun Chin, Sejoong Kim

**Affiliations:** 1 Division of Nephrology and Hypertension, Department of Internal Medicine, Inha University College of Medicine, Incheon 22332, Korea; kpkidney@inha.ac.kr; 2 Research Center for Materials Analysis, Korea Basic Science Institute, Daejeon 34133, Korea; bjeong@kbsi.re.kr; 3 Department of Internal Medicine, Seoul National University Bundang Hospital, Seongnam 13620, Korea; yunmi1202@hanmail.net (Y.-M.L.); she0817@naver.com (H.-E.S.); jyryu1022@gmail.com (J.-Y.R.); no1seokwoo@gmail.com (S.P.); jcj0425@empal.com (J.C.J.); mednep@snubh.org (H.J.C.); 4 Department of Biomedical Sciences, Seoul National University College of Medicine, Seoul 03080, Korea; 5 Department of Internal Medicine, Seoul National University College of Medicine, Seoul 03080, Korea

**Keywords:** contrast-induced nephropathy, kidney-on-a-chip, acute kidney injury, contrast media

## Abstract

Increased viscosity of concentrated contrast media (CM) in the renal tubules can perturb renal hemodynamics and have a detrimental effect on tubular epithelial cells. However, the effects of viscosity on contrast-induced nephropathy (CIN) remain poorly understood. Conventional in vitro culture studies do not reflect the rheological properties of CM. Therefore, we investigated the effects of CM viscosity on renal tubules using a kidney-on-a-chip and two different types of CM. Renal proximal tubule epithelial cells (RPTEC) were cultured in a three-dimensional microfluidic culture platform under bidirectional fluid shear stress. We treated the RPTEC with two types of CM: low- (LOCM, iopromide) and iso-osmolar contrast media (IOCM, iodixanol). Renal tubular cell injury induced by LOCM and IOCM was examined under different iodine concentrations (50–250 mgI/mL) and shear-stress conditions. LOCM showed a significant dose-dependent cytotoxic effect, which was significantly higher than that of IOCM under static and low-to-moderate shear stress conditions. However, high shear-stress resulted in reduced cell viability in IOCM; no difference between IOCM and LOCM was found under high shear-stress conditions. The cytotoxic effects were pronounced at a mean shear stress of 1 dyn/cm^2^ or higher. The high viscosity of IOCM slowed the fluid flow rate and augmented fluid shear-stress. We suggest an alternative in vitro model of CIN using the three-dimensional kidney-on-a-chip. Our results indicate a vital role of viscosity-induced nephrotoxicity under high shear-stress conditions, contrary to the findings of conventional in vitro studies.

## 1. Introduction

Contrast-induced nephropathy (CIN) is a major health problem that manifests as an abrupt decline in renal function after iodine contrast exposure. CIN is known as the third most common cause of hospital-acquired acute kidney injury [1]. CIN contributes to chronic kidney disease (CKD) progression and prolonged length of hospital stay. Moreover, the rapidly increasing use of computed tomography scans or interventions using contrast agents, such as angiography, is raising concerns regarding CIN. In addition, there are limited options for the prevention and treatment of CIN, including fluid therapy and avoidance of additional nephrotoxins. Potential pathogenic mechanisms of CIN include vasoconstriction, medullary hypoxia, direct cytotoxicity, oxidative stress, and increased tubular fluid viscosity [2]. Previous experimental studies have mainly focused on direct tubular toxicity, vasoconstriction, and oxidative stress following contrast exposure [3].

Many researchers have linked the physicochemical properties of iodinated contrast materials to CIN. The administered volume, osmolality, and viscosity of contrast media (CM) may be related to nephrotoxicity [4]. Indeed, high-osmolar ionic CM is no longer used because of the high risk of adverse drug reactions and renal toxicity [5]. Currently, two types of contrast agents are widely used in clinical practice: high-viscosity iso-osmolar contrast media (IOCM) and low-viscosity low-osmolar contrast media (LOCM). IOCM has an osmolality similar to that of blood (290 mOsm/kg), while LOCM has a higher osmolality (600–900 mOsm/kg) than IOCM. Some studies have suggested the superiority of IOCM over LOCM in the prevention of CIN [6,7]. However, there was no difference in the overall risk of CIN according to the type of CM in meta-analyses [8,9]. It still remains unclear which of these two types of CM is better for renal safety.

To date, the effect of CM viscosity on renal function has not been fully investigated due to the difficulty in experimental methodology. Accurately controlling and measuring fluid viscosity and shear stress is challenging in conventional experimental designs. Under in vivo conditions, the high viscosity of IOCM can be harmful to renal function. IOCM is further concentrated in the renal medulla with increasing viscosity, particularly in dehydrated individuals, which increases intraluminal pressure and decreases the glomerular filtration rate (GFR) [10]. Nevertheless, previous in vitro studies in static conditions showed that LOCM has more cytotoxic effects than IOCM [11]. However, this static condition does not reflect the rheological effects of CM. Given that animal models for CIN are not well established and clinical trials are much harder to implement due to high cost and ethical issues [12], alternative experimental approaches are warranted. In the field of nephrology, organ-on-a-chip technology is gradually being utilized for physiological in vitro disease models [13]. One of the key features of the organ-on-a-chip is the exposure of cells to easily controlled microfluidic conditions, which could enable rheological studies. Therefore, organ-on-a-chip experiments could be an alternative approach to bridge the gap between the in vitro and in vivo findings in CIN. In the present study, we aimed to evaluate the nephrotoxic effects of the rheological factors of LOCM and IOCM using a kidney-on-a-chip platform.

## 2. Materials and Methods

### 2.1. Cell Culture and Microfluid Device

**Figure 1:**
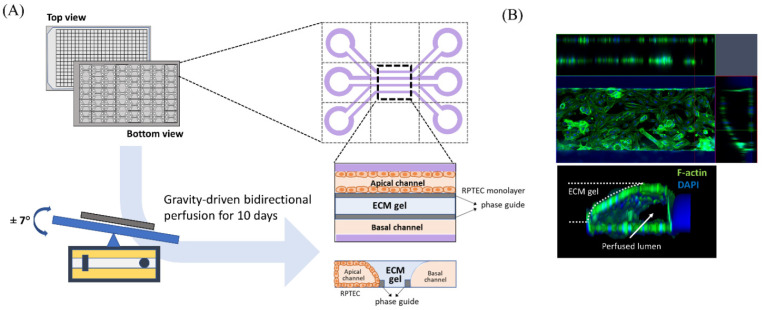
Schematic diagram of modeling a three-dimensional (3D) tubular structure in the three-lane OrganoPlate. (**A**) The three-lane OrganoPlate consists of 40 microfluidic channel units based on a 384-well plate format. After gravity-driven bidirectional perfusion for 10 days, a 3D tubular structure is formed. The basal channel is filled with culture media. (**B**) Image of 3D reconstruction of a single unit of the kidney-on-a-chip. ECM, extracellular matrix; DAPI, 4′,6-diamidino-2-phenylindole.

Human renal proximal tubule epithelial cells (RPTEC; CC-2553, Lonza, Basel, Switzerland) were purchased and used to evaluate the nephrotoxicity of iodinated CM. RPTEC were cultured in renal epithelial growth medium (REGM CC-3191, Lonza) supplemented with 0.5% FBS (CC-4127, Lonza) and GA-1000 (30 mg/mL gentamicin and 15 µg/mL amphotericin). We used a three-lane OrganoPlate (Mimetas BV, 4003 400B) with a channel width of 400 μm and height of 220 μm to construct three-dimensional tubular structures (Figure 1A). Before cell seeding, 1.6 μL of collagen 1 (AMSbio cultrex, 3447-020-01, 4 mg/mL) was injected into the middle inlet of the chip with 100 mM HEPES (15630, Life Technologies, CA, USA) and 3.7 mg/dL NaHCO_3_ (S5761, Sigma, St. Louis, MO, USA), and the chip was subsequently incubated at 37 °C overnight. RPTEC were seeded at 2 × 10^7^/mL into each upper inlet of the plate. To facilitate cell attachment to the extracellular matrix, we maintained the inclination of the chip at 70° for 4 h before placement on an interval rocker. RPTEC were perfused at an angle of 7° and at intervals of 8 min using a rocker at 37 °C in a humidified 5% CO_2_ atmosphere. Under our experimental conditions, the cells reached confluence 10 days after rocking perfusion. Before administration of the test solutions, we examined whether the three-dimensional tubular structure was well formed within the chips. Three-dimensional reconstructed fluorescent images revealed a well-demarcated tubular lumen with a confluent epithelial monolayer (Figure 1B). For comparison with static culture conditions, RPTEC was also cultured in 96-well plates without fluid shear stress in a 37 °C incubator with 5% CO_2_.

### 2.2. Chemicals and Reagents

**Figure 2:**
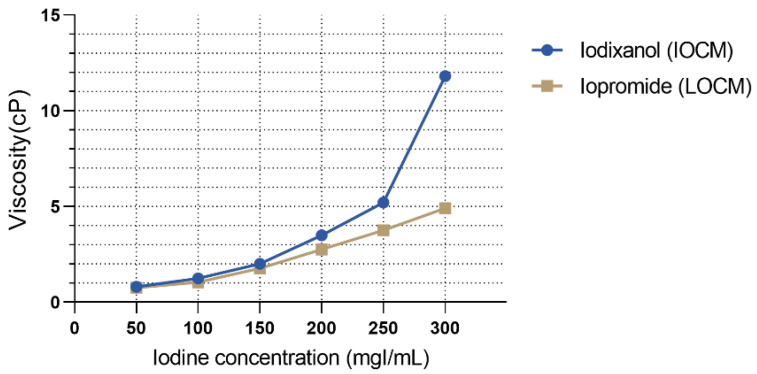
Viscosity of contrast media according to different iodine concentrations.

The test solutions were IOCM iodixanol (Visipaque 320 mgI/mL, GE Healthcare, Chicago, IL, USA), LOCM iopromide (Ultravist 300 mgI/mL, Bayer Healthcare, Berlin, Germany), 6% hydroxyethyl starch 130/0.4 (viscosity control; Volulyte, Fresenius Kabi, Bad Homburg, Germany), and 15% mannitol (osmolality control; JW Pharmaceutical, Seoul, Korea). The characteristic parameters of the test solutions are listed in Table 1. To simulate the urine concentration process, we tested different CM concentrations. The dynamic viscosity of CM specimens according to the preselected iodine concentrations was measured with an SV-10 viscometer (A&D, Japan) at a temperature of 37 °C with three replicates per group. As shown in Figure 2, the viscosity of each contrast agent increased exponentially with iodine concentration. The viscosities of LOCM and IOCM differed substantially at high iodine concentrations. Cell viability was determined using the CCK-8 assay (Dojindo Laboratories, Kumamoto, Japan) performed according to a standard protocol, and the absorbance of the samples was measured at 450 nm using a fluorescence microplate reader (Gemini EM, Molecular Devices, San Jose, CA, USA). Cells were detached from the OrganoPlate using Accutase (A6964, Sigma) and lysed in ice-cold cell lysis buffer (9803, Cell Signaling, Danvers, MA, USA). Protein lysates were centrifuged at 14,000× *g*, 4 °C for 10 min, and the supernatant was collected. Protein extracts were quantified using a Pierce BCA Protein Assay kit (23227, Thermo Fisher Scientific, Rockford, IL, USA) and separated with 10% polyacrylamide gel electrophoresis (PAGE) followed by transfer to polyvinylidene fluoride membranes (1704156, Bio-Rad, Hercules, CA, USA). The membrane was incubated with primary antibodies against GAPDH (Santa Cruz, 47724), Akt (Cell Signaling, 9272), phospho-Akt (p-Akt; Ser473; 9271, Cell Signaling), extracellular signal-regulated kinase1/2 (Erk1/2; 9102, Cell Signaling), and phospho-Erk1/2 (p-Erk1/2; Thr202/Tyr204; 9101, Cell Signaling), followed by the corresponding secondary antibodies (7074 and 7076, Cell Signaling). Each protein band was developed using SuperSignal West Pico PLUS Chemiluminescent Substrate (34577, Thermo Fisher Scientific) and detected using the ChemiDoc Touch Imaging System (Bio-Rad).

### 2.3. Experimental Model

In the present study, we investigated CM-induced nephrotoxicity under shear-stress conditions using kidney-on-a-chip models compared to that under static conditions. RPTEC was treated with varying iodine concentrations of IOCM and LOCM, which reflect renal concentrating process. Because IOCM and LOCM have different osmolalities and viscosities, we compared the nephrotoxic effects of each contrast agent in terms of physicochemical stress and performed mathematical simulations. IOCM and LOCM were used to evaluate the effects of high viscosity and osmolality on renal tubular cells, respectively. We tested each experimental group under different shear-stress conditions. Fluid shear-stress in vivo in human proximal tubules is estimated to be 0.1–1 dyn/cm^2^ [14,15,16]; however, it was applied differently depending on the experimental conditions [17]. In a rectangular pipe-shaped channel of the OrganoPlate, the fluid shear-stress (τ) can be calculated as $\tau =\frac{6µQ}{{\mathrm{bh}}^{2}}$, where b and h represent the width and height of the channel, respectively, Q represents the flow rate, and μ denotes the fluid viscosity. The fluid shear-stress is approximately 0.13 dyn/cm^2^ when the rocker is set at an inclination of ±7° and an interval of 8 min [18]. Given that a single nephron glomerular filtrate directly reaches the initial part of the proximal tubule, a single nephron glomerular filtration rate (SNGFR) can be used for the calculation of shear stress. The SNGFR of healthy adults has been reported to be 80 ± 40 nL/min [19], and the diameter of the proximal tubule has been reported to be ~60 μm [20,21]. The fluid shear-stress at the proximal tubular wall can be calculated as $\tau =\frac{4µQ}{{\pi R}^{2}}$ under the assumption of a laminar, incompressible Newtonian flow of urine filtrate through a cylindrical tube, where R represents the radius of the proximal tubule. Since about 60–70% of the filtered water and sodium is reabsorbed in the proximal tubule [22], fluid shear-stress in the human proximal tubule ranges from approximately 0.44 (initial part) to 0.13 (distal part) dyn/cm^2^ (at 37 °C), which is comparable to that in the OrganoPlate. CKD is accompanied by a progressive loss of functioning glomeruli in the kidneys. Depending on renal disease, nephron loss induces an increase in the SNGFR of remaining nephrons [23]. These phenomena are particularly observed in post-nephrectomy animal models [24]. To mimic progressive nephron loss, we designed different mean flow rate conditions with varying rocker intervals (8 min, 4 min, and 2 min) applied to the chips.

### 2.4. Mathematical Simulation

Mathematical simulation was performed using Python (Python Software Foundation, Wilmington, DE, USA) and gnuplot to examine the flow rate and shear stress in the upper channel of the chip. The test fluids, including iodinated CMs, were regarded as Newtonian fluids with constant viscosity and incompressibility. Parameters such as the dimensions of the OrganoPlate provided by the manufacturer were used. Mass density and dynamic viscosity at 37 °C were used for the calculations. Different viscosities of test fluids and rocking conditions were also considered. The shear stress and fluid flow rate at each time point during a single cycle of rocking were calculated. The time-averaged shear-stress and flow rate were calculated from the average of each value over multiple cycles of bidirectional flows.

### 2.5. Image and Statistical Analysis

Three-dimensional images were reconstructed using the ImageJ software (NIH, USA). Graph plotting and data analysis were performed using GraphPad Prism 9 (GraphPad Software, Inc., San Diego, CA, USA). Data are presented as the mean ± standard error of the mean (SEM). Each experiment was performed and analyzed in triplicate. Differences between experimental groups were compared using ANOVA and Bonferroni *t*-tests. *p* values < 0.05 were considered statistically significant.

## 3. Results

### 3.1. Mathematical simulation

**Figure 3:**
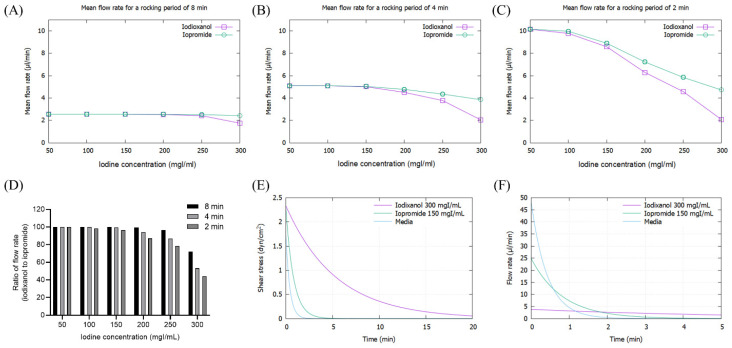
Mathematical simulation of fluid flow rates and shear stresses of iopromide and iodixanol. (**A**) Mean flow rate with a rocker interval of 8 min; (**B**) mean flow rate with a rocker interval of 4 min; (**C**) mean flow rate with a rocker interval of 2 min; (**D**) ratio of flow rate (iodixanol to iopromide); (**E**) fluid shear-stress during a single cycle of rocking. The concentrations of iodixanol and iopromide were selected on the assumption that IOCM is approximately two times more concentrated than LOCM through the renal tubule. (**F**) Fluid flow rate during a single cycle of rocking.

As the viscosity of CM increased with concentration, the mean flow rate of CM decreased, particularly at high iodine concentrations of more than 200 mg/dL, which was pronounced at 2 min rocking intervals (Figure 3A–C). The flow rate of IOCM was substantially reduced due to its relatively higher viscosity compared to LOCM. The difference in flow rate between IOCM and LOCM was apparent at higher iodine concentrations and shorter rocking intervals (Figure 3D). Notably, the flow rate of IOCM at an iodine concentration of 300 mgI/mL and 2 min intervals was less than half of that of LOCM. Since shear stress is linearly proportional to the product of fluid viscosity and flow rate, the peak shear-stress is theoretically not different between IOCM and LOCM. However, time-averaged shear-stress was greater in IOCM than in LOCM due to the slower fluid flow rate (Supplementary Figures S1 and S2). This indicates that IOCM is retained longer than LOCM, which also implies longer exposure to CM-induced nephrotoxicity. In the human kidney, urinary flow is not bidirectional; thus, we additionally examined the shear stress and fluid flow-rate versus time within a single cycle (Figure 3E,F). Depending on the viscosity of the test fluid, time durations of shear stress above 1 dyn/cm^2^ were 0.23 min (media), 0.67 min (iopromide), and 4.52 min (iodixanol; Figure 3E). Peak flow rates were 48.08 μL/min (media), 24.82 μL/min (iopromide), and 3.82 μL/min (iodixanol; Figure 3F). Iodixanol flows more slowly than iopromide and causes a higher shear-stress for a longer duration.

### 3.2. Modeling of Contrast-Induced Nephropathy in Kidney-on-a-Chip

**Figure 4:**
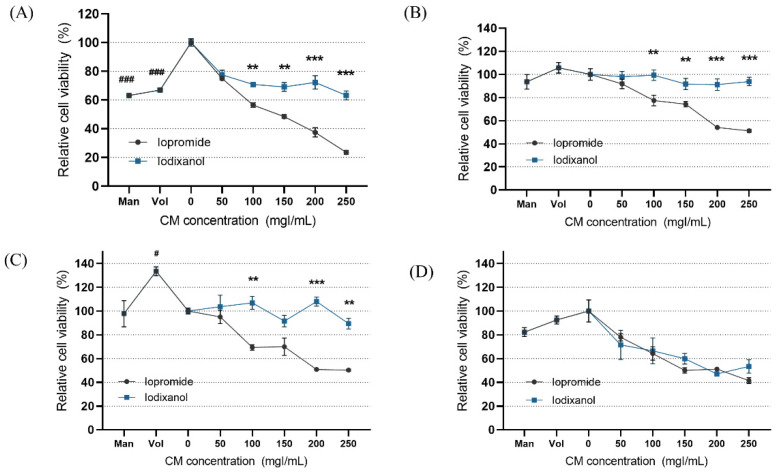
Cell viability according to the different fluid viscosity and shear-stress conditions at (**A**) static condition, (**B**) a rocker interval of 8 min, (**C**) a rocker interval of 4 min, and (**D**) a rocker interval of 2 min. CM—contrast media; Man—mannitol; HES—hydroxyethyl starch; Con—negative control group. Data are represented as the mean ± SEM. ^###^ *p* < 0.001 versus control (0 mgI/mL); ^#^ *p* < 0.05 versus control (0 mgI/mL); ** *p* < 0.01 versus iopromide group of the same iodine concentration; *** *p* < 0.001 versus iopromide group of the same iodine concentration.

**Figure 5:**
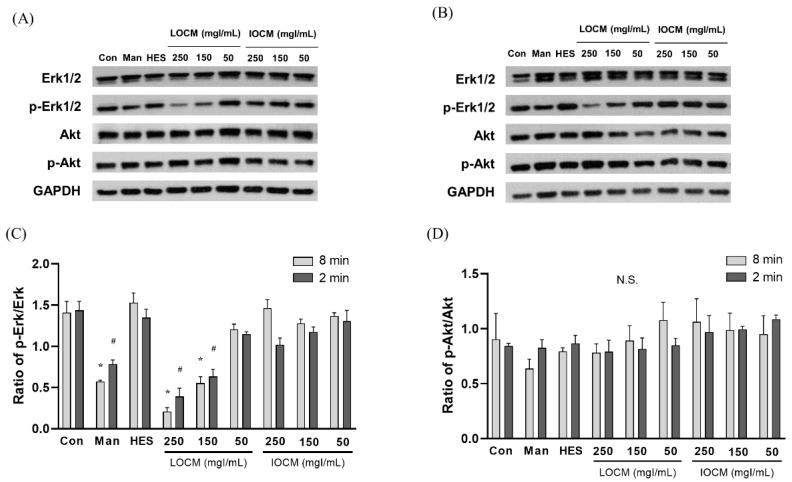
Representative immunoblots of Akt, p-Akt, Erk1/2, p-Erk1/2, and β-actin at (**A**) a rocker interval of 8 min and (**B**) a rocker interval of 2 min. (**C**) Ratio of p-Erk1/2/Erk1/2. (**D**) Ratio of p-Akt/Akt. Data are represented as the mean ± SEM. * *p* < 0.05 versus control of 2 min interval, ^#^ *p* < 0.05 versus control of 8 min interval. LOCM—low-osmolar contrast media; IOCM—iso-osmolar contrast media; Con—control; Man—mannitol; HES—hydroxyethyl starch; N.S.—nonsignificant.

In the static condition, the overall cytotoxicity was higher for both LOCM and IOCM compared to the control (Figure 4). In particular, LOCM showed a marked dose-dependent cytotoxic effect, which was significantly higher than that of IOCM at concentrations of 100 mgI/mL or higher. This finding was consistent with a previous study [11]. Under shear-stress conditions using bidirectional rocker perfusion at 8 min intervals, there was no difference in cell viability between IOCM and the control across all iodine concentrations. However, LOCM induced greater cytotoxicity than IOCM. LOCM significantly decreased cell viability in the range of 100–250 mgI/mL, with corresponding osmolalities of 393, 447, 501, and 555 mOsm/kg, whereas those of IOCM ranged from 286 to 290 mOsm/kg. These patterns remained unchanged at 4 min rocking intervals; LOCM was more cytotoxic than IOCM at the same range of concentrations. Interestingly, increased cell viability was observed in some high-viscosity experimental groups, such as the HES and IOCM groups. However, at 2 min rocking intervals, there was no difference in cytotoxicity between LOCM and IOCM. Both LOCM and IOCM induced significant cytotoxicity at concentrations of 150 mgI/mL or higher. Mean shear-stress at the ranges showing cytotoxic effects was estimated from 0.89 dyn/cm^2^ (iodixanol concentration of 150 mgI/mL) to 1.23 dyn/cm^2^ (iodixanol concentration of 250 mgI/mL) from the mathematical simulation. The corresponding mean flow rate of iodixanol ranged from 8.59 μL/min to 2.09 μL/min. Under the same experimental conditions, we performed Western blot analysis of signaling molecules reported to be associated with CIN, including Erk1/2, and Akt (Figure 5) [25]. During the 8 min rocking interval, LOCM significantly induced a decrease in the phosphorylation of Erk1/2, especially at high concentrations. A similar pattern was observed when cells were treated with the high-osmolar agent mannitol. However, there was no change in the expression of these molecules in IOCM groups. This pattern was similar when applying the 2 min rocking interval. LOCM inhibited the phosphorylation of Erk1/2 in a dose-dependent manner. IOCM did not induce any significant changes in the expression of signaling molecules at any concentration.

## 4. Discussion

In the present study, we suggest an alternative in vitro model of CIN using a three-dimensional proximal tubule-on-a-chip. Renal tubular cell injury induced by IOCM and LOCM was examined under different iodine concentrations and shear-stress conditions, which represent urine concentrations depending on the hydration status and SNGFR. The high viscosity of the concentrated IOCM noticeably slowed the tubular flow rate and increased the mean shear-stress levels. We identified significant cytotoxic effects from the increased viscosity of IOCM within a three-dimensionally perfused channel, which was not apparent under low-shear-stress conditions. LOCM was cytotoxic, even under static or low-shear-stress conditions, and the cytotoxicity was possibly mediated by Erk1/2 inactivation. However, nephrotoxicity was comparable between LOCM and IOCM under high-shear-stress conditions.

To date, researchers have suggested that the high viscosity of CM is one of the pathogenic mechanisms of CIN. In particular, IOCM has physiological osmolality at the cost of increased viscosity, and thus, its high viscosity could have detrimental effects on the kidney. However, the rheological aspects of CM have not been fully addressed in the pathophysiology of CIN. A previous in vitro study revealed that LOCM is more cytotoxic than IOCM under static conditions, which is inconsistent with clinical data [11]. The implementation of the microenvironment in a single nephron was not feasible using conventional in vitro experiments. The physicochemical properties of CM have been evaluated using indirect methods, such as dialysis [26]. We constructed the CIN-on-a-chip using the OrganoPlate platform, which contains an extracellular matrix and forms a 3D perfused tubular structure, mimicking an in vivo environment [18]. Functional studies of transporters, biomarkers, and gene expression using the OrganoPlate have already been tested and validated [27]. Given that renal tubular epithelial cells are continuously exposed to the fluid shear-stress of urine filtrate [28], kidney-on-a-chip may be an indispensable tool for the rheological study of renal diseases. To the best of our knowledge, this is the first study on CIN using an organ-on-a-chip platform.

Overall, our findings showed nephrotoxic effects of CM under shear-stress conditions which were distinct from those under static conditions. Under static conditions, higher cytotoxicity was observed even at low concentrations of CM compared to that of the control, which is not seen under in vivo conditions and in clinical settings. Since CIN mainly occurs when high-dose CM are used in patients with risk factors [29], our results under shear-stress conditions are more similar to the clinical situations. These findings may indicate the beneficial effects of fluid shear-stress within physiological levels. Shear stress is reported to activate Nrf2 expression in the renal proximal tubules, resulting in a protective role in CIN [30,31]. However, our findings also highlight the critical role of the high viscosity of IOCM in CIN. Increased viscosity of CM could contribute to tubular cell damage through flow-derived stresses such as tubular stretching, increased shear stress, and transmural pressure [32]. Similarly, renal tubular injury in urinary tract obstruction models is also mediated mainly through cellular stretching and altered shear-stress [33]. Fluid shear-stress beyond physiological levels could cause renal epithelial damage. Although the exact mechanisms are yet to be elucidated, there has been some evidence related to the harmful effects of increased shear-stress on the cells. Studies on microfluidic platforms suggest that excessive shear-stress has detrimental effects on cells by detaching or disrupting the cytoskeleton [29,30]. Exposure of HK2 cells to high shear-stress (5 dyne/cm^2^) for 48 h caused structural derangement, including the loss of tight junctions, adherent junctions, and primary cilia [17]. Therefore, most microfluidic studies with renal tubular epithelial cells have used a physiological shear-stress of 0.2–1 dyn/cm^2^. As observed in our findings, viscosity-induced cytotoxicity was pronounced at a mean shear-stress of 1 dyn/cm^2^ or higher. Additionally, a study by Garvin et al. suggested the vital role of tubular cell stretching in producing reactive oxygen species [34], which is a key pathogenesis in CIN.

**Figure 6:**
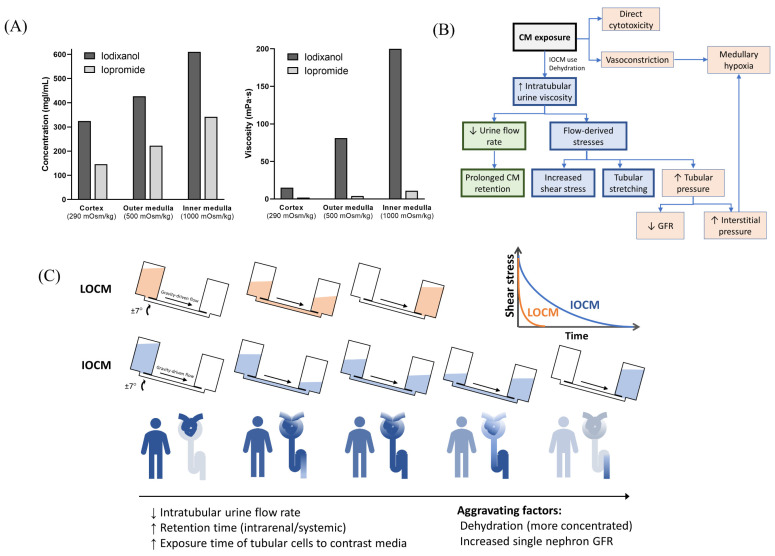
Overview of the proposed models of contrast-induced nephropathy. (**A**) Iso-osmolar contrast media are twice as concentrated as low-osmolar contrast media and have a much greater viscosity. Data from Jost et al. [26]. The concentration and viscosity of CM are obtained from an in vitro dialysis model using polyethylene glycol solution with different osmolalities (290, 500, and 1000 mOsm/kg, which represents the mean osmolality of the renal cortex, outer medulla, and inner medulla, respectively). (**B**) Overall mechanisms of contrast-induced nephropathy. (**C**) Comparison of iso-osmolar and low-osmolar contrast media in our model. CM—contrast media; LOCM—low-osmolar contrast media; IOCM—iso-osmolar contrast media; GFR—glomerular filtration rate.

With a typical dose of CM, the plasma concentration is less than 20 mgI/mL [35]. However, CM are not reabsorbed in the renal tubules, and their intraluminal concentration increase along the renal tubules, with a peak concentration up to 100 times higher than that in plasma [36]. IOCM are approximately two times more concentrated due to their lower osmolality, which further exponentially increases urine viscosity (Figure 6A) [26]. In our mathematical simulation, the time constant of exponential decay for the fluid flow-rate is proportional to the fluid viscosity; therefore, it is much greater with IOCM. As a result, IOCM is excreted more slowly than LOCM, exerting high levels of shear stress on the tubular cells for a longer duration (Figure 6B,C). We showed that when assuming that the iodixanol and iopromide concentrations at the corticomedullary junction are 300 mgI/mL and 150 mgI/mL, respectively, the peak flow rate of iodixanol was six times slower than that of iopromide, and the time during which the shear stress level is above 1 dyn/cm^2^ was also seven times longer with iodixanol than with iopromide. These findings are in line with the results of some in vivo studies that related highly viscous CM to increased tubular pressure, prolonged CM retention, and subsequent decreased GFR and hypoxia [10]. A micropuncture study in rats by Ueda et al. showed that the viscosity of IOCM (iotrolan) was significantly higher than that of other LOCMs with slower urinary excretion [37]. Iotrolan also induced high intratubular hydrostatic pressure for a longer duration than LOCMs [38]. The high viscosity of iodixanol induces prolonged reduction in medullary oxygenation [39], which is a key feature of CIN. Nevertheless, these animal studies require sophisticated techniques and lack reproducibility; thus, similar findings have only been identified in a few studies.

Urine viscosity can vary significantly with urine volume, which is closely associated with an individual’s hydration status. The effect of dehydration on renal concentration is more pronounced when using IOCM [10]. We assumed that CM was moderately to highly concentrated in the renal tubules. In addition, given that patients with CKD are at a greater risk of CIN, different shear-stress conditions were investigated to reflect the effect of nephron loss and increased SNGFR. Our findings indirectly showed that highly concentrated CM and nephron loss could contribute to increased shear-stress and renal toxicity, which is consistent with the results of a uninephrectomy mouse model [17]. We treated RPTEC with CM for 24 h, which also reflected the CKD condition. Patients with CKD usually show a longer retention of CM. Cortical retention of CM was observed in patients with CKD 24 h after CM administration, which was more pronounced in patients later diagnosed with CIN [40]. Conventionally, the high osmolality of CM is considered a major determinant of CIN. However, the role of osmolality in CIN pathogenesis has not been clearly determined. Rather, LOCM can confer beneficial effects on tubular cells by inducing osmotic diuresis and subsequently lowering urine viscosity. Indeed, LOCM causes a two-fold higher urine output than IOCM due to osmotic diuresis [41]. In this regard, the higher osmolality of LOCM relative to IOCM may serve as a protective factor against viscosity-induced renal toxicity.

Additionally, we evaluated some pathway molecules that have been reported to be linked to CIN. Akt and Erk1/2 are among the molecules involved in cell survival and proliferation [25]. A previous in vitro study of CIN showed that both IOCM and LOCM induced dephosphorylation of Akt and Erk1/2 [25]. In particular, IOCM caused a greater decrease in p-Erk 1/2 in HK2 cells. However, we revealed that decreased phosphorylation of Erk1/2 was only observed in the LOCM group under shear-stress conditions, and there was no difference in p-Akt levels between groups. Although our study was not designed to evaluate the exact mechanistic pathways of CIN, our findings possibly suggest that osmolar stress is associated with dephosphorylation of Erk1/2 or viscosity-related stress induces the activation of Erk1/2. Mechanical stretching of renal proximal tubular cells activates Erk1/2 signaling [42], and high-osmolar CM diatrizoate causes greater dephosphorylation of Erk1/2 compared to iopromide [43].

Collectively, viscosity-mediated nephrotoxicity was prominent under high shear-stress conditions. We found no difference between IOCM and LOCM under high fluid-shear-stress, which was similar to the clinical data. These findings suggest that the risk of CIN does not differ between IOCM and LOCM in patients with CKD. Even in a study showing a better effect of IOCM on renal toxicity, superiority of IOCM was not significant in the subgroup with lower GFR (<45 or 60 mL/min/1.73 m^2^) [44]. In this context, the kidney-on-a-chip microfluidic platform could be a potential alternative in vitro model, which can provide easier control of various physical parameters.

Our study has some limitations. This study highlights the critical role of the rheological effects of CM in terms of tubulodynamics. However, detailed mechanisms that mediate viscosity-induced tubular injury still remain elusive. More sophisticated methodology is warranted to determine the main contributing factors to viscosity-related injury. High viscosity also affects the hemodynamics in peritubular vessels. Therefore, extended microfluidic models combining vascular compartments with tubular counterparts would be useful. In addition, the limitations of the assumptions of the experimental design should be considered. We mathematically regarded the renal tubule as a rigid channel; however, it was highly elastic and deformable. In addition, the bidirectional pulsatile flow used in our model was not the same as the unilateral fluid flow in the renal tubules.

## 5. Conclusions

We demonstrated the application of a three-dimensional kidney-on-a-chip model to evaluate the physicochemical factors of CM in CIN. Overall, our CIN model under shear-stress conditions reliably reflected in vivo situations. Our results highlight the role of viscosity-induced nephrotoxicity under high-shear-stress conditions and are quite different from the findings of conventional in vitro studies. LOCM showed higher cytotoxicity than IOCM in a dose-dependent manner under physiological shear-stress conditions. However, under high-shear-stress conditions, IOCM caused renal tubular cell damage comparable to that caused by LOCM, decreasing the urine flow rate and exposing cells to high shear-stress for a longer duration.

## Figures and Tables

**Table 1:** Characteristics of test solutions.

	Iodixanol (Visipaque™)	Iopromide (Ultravist™)	6%HES (VOLULYTE^®^)	15% Mannitol	Negative Control (Culture Media)
Iodine concentration (mgI/dL)	320	290	-	-	-
Osmolality (mOsm/kg)	290	607	283	823	288
Viscosity (cP, at 37 °C)	11.8	4.9	2	1	0.8

Values other than those of the control were based on the data provided by the manufacturers. HES—hydroxyethyl starch.

## Supplementary Materials

The following supporting information can be downloaded at: https://www.mdpi.com/article/10.3390/mi13050688/s1, Figure S1: Mathematical simulation of bidirectional flows in iopromide groups; Figure S2: Mathematical simulation of bidirectional flows in iodixanol groups.
