# Supplementary material for: Three-Dimensional Kidney-on-a-Chip Assessment of Contrast-Induced Kidney Injury: Osmolality and Viscosity
Source: Micromachines (Basel). 2022 Apr 28;13(5):688. doi: 10.3390/mi13050688 (PMC9146534; doi:10.3390/mi13050688)
Supplement: Supplementary file 1 [file micromachines-13-00688-s001.zip › micromachines-1673658-supplementary.pdf]

Supplementary Materials

Three-Dimensional Kidney-on-a-Chip Assessment of Con-trast-induced Kidney Injury: Osmolality and Viscosity

Kipyo Kim [1], Beomgyun Jeong [2], Yun-Mi Lee [3], Hyung-Eun Son [3], Ji-Young Ryu [3], Seokwoo Park [3,4], Jong Cheol Jeong [3], Ho Jun Chin [3,5], Sejoong Kim [3,5,*]

[1] Division of Nephrology and Hypertension, Department of Internal Medicine, Inha University College of Medicine, Incheon 22332, Korea; kpkidney@inha.ac.kr
[2] Research Center for Materials Analysis, Korea Basic Science Institute, Daejeon 34133, Korea; bjeong@kbsi.re.kr
[3] Department of Internal Medicine, Seoul National University Bundang Hospital, Seongnam 13620, Korea; yunmi1202@hanmail.net (Y.-M.L.); she0817@naver.com (H.-E.S.); jyryu1022@gmail.com (J.-Y.R.); no1seokwoo@gmail.com (S.P.); jcj0425@empal.com (J.C.J.); mednep@snubh.org (H.J.C.)
[4] Department of Biomedical Sciences, Seoul National University College of Medicine, Seoul 03080, Korea
[5] Department of Internal Medicine, Seoul National University College of Medicine, Seoul 03080, Korea

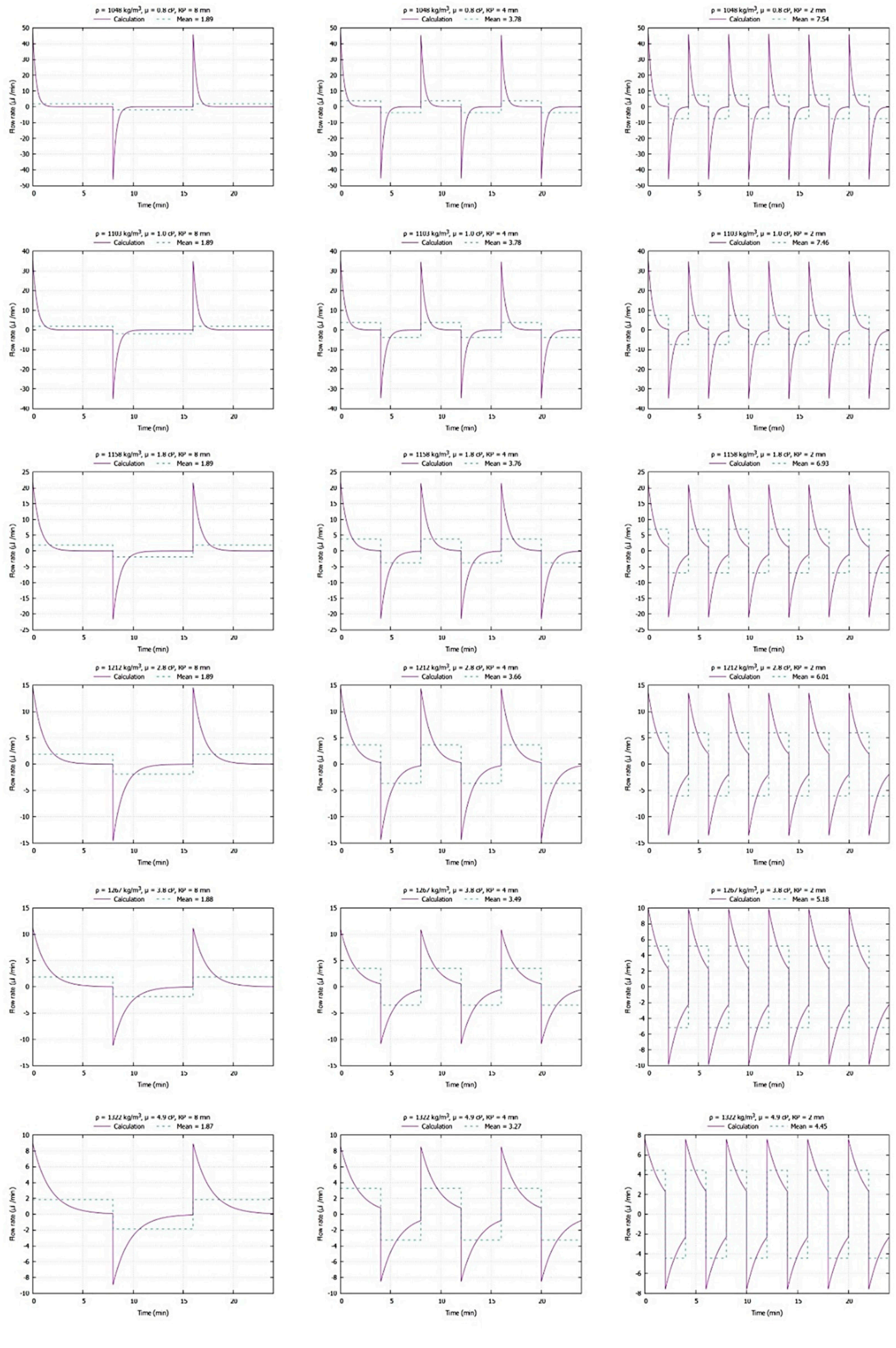

Figure S1. Mathematical simulation of bidirectional flows in iopromide groups.

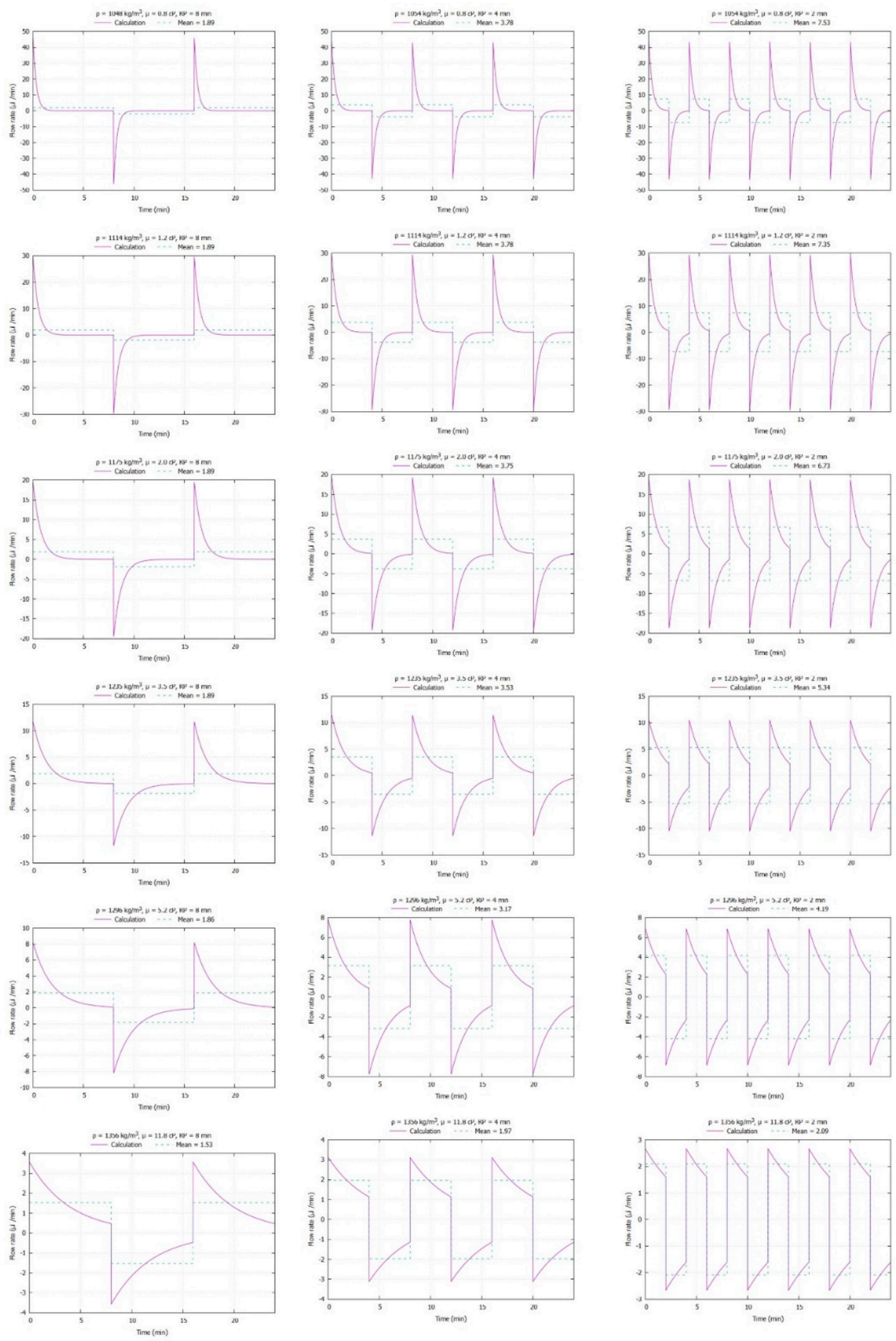

Figure S2. Mathematical simulation of bidirectional flows in iodixanol groups.
